# Impact of Capsulectomy Type on Post-Explantation Systemic Symptom Improvement: Findings From the ASERF Systemic Symptoms in Women-Biospecimen Analysis Study: Part 1

**DOI:** 10.1093/asj/sjab417

**Published:** 2021-12-16

**Authors:** Caroline Glicksman, Patricia McGuire, Marshall Kadin, Marisa Lawrence, Melinda Haws, Jill Newby, Sarah Ferenz, James Sung, Roger Wixtrom

**Affiliations:** Hackensack Meridian School of Medicine, Nutley, NJ, USA; Washington University, St. Louis, MO, USA; Department of Pathology and Laboratory Medicine, Warren Alpert Medical School, Brown University, Providence, RI, USA; Department of Plastic Surgery, Vanderbilt University, Nashville, TN, USA; School of Psychology, University of New South Wales, Sydney, Australia; Chicago Medical School, North Chicago, IL, USA; Department of Pathology and Laboratory Medicine, Warren Alpert Medical School, Brown University, Providence, RI, USA

## Abstract

**Background:**

Breast Implant Illness (BII) is a term used to describe a variety of symptoms by patients with breast implants for which there are no abnormal physical or laboratory findings to explain their symptoms. There currently exists a difference of opinion among clinicians and patients concerning the diagnosis and treatment of patients self-reporting BII.

**Objectives:**

The first aim of this study was to determine if there is a valid indication for “en bloc” capsulectomy in patients self-reporting BII and if the type of capsulectomy performed alters long-term symptom improvement. The second goal was to identify any clinical laboratory differences between the cohorts. This study was funded by the Aesthetic Surgery Education and Research Foundation (ASERF).

**Methods:**

A prospective blinded study enrolled 150 consecutive subjects divided equally into 3 cohorts: (A) women with systemic symptoms they attribute to their implants who requested implant removal; (B) women with breast implants requesting removal or exchange who do not have symptoms they attribute to their implants; and (C) women undergoing cosmetic mastopexy who have never had any implanted medical device. The subject’s baseline demographic data and a systemic symptoms survey, including PROMIS validated questionnaires, was obtained before surgery and at 3-6 weeks, 6 months, and 1 year. Blood was collected from all 3 cohorts and implant capsules were collected from Cohorts A and B.

**Results:**

150 patients were enrolled between 2019-2021. Follow-up at 3-6 weeks for all 3 cohorts was between 98%-100%, 78%-98% at 6-months, and 1 year data is currently at 80%. The type of capsulectomy; intact total, total, or partial all showed similar symptom improvement with no statistical difference in the reduction of symptoms based on the type of capsulectomy.

**Conclusions:**

This study addresses one of the most discussed questions by plastic surgeons, patients, their advocates, and social media. The findings show that patients who self-report BII demonstrate a statistically significant improvement in their symptoms after explantation and that this improvement persists for at least 6 months. This improvement in self-reported systemic symptoms was seen regardless of the type of capsulectomy performed.

**Level of Evidence: 2:**

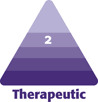

Possible associations between systemic illness and breast implants have been hypothesized for over half a century. Initial reports of “Human Adjuvant Disease” appeared in the literature based upon complications associated with the injection of paraffin, processed petroleum, and adulterated liquid silicone either directly into the breasts or the use of early generation silicone filled elastomer implants.^1,2^ Shoenfeld first coined the term ASIA Syndrome (Autoimmune Syndrome Induced by Adjuvants) in 2011.^3^ This syndrome continues to be discussed in the world literature though most publications are opinions, anecdotal reports, retrospective chart reviews, or editorials. ASIA remains a syndrome without a distinct causative agent and no clear temporal association, often with decades between initial exposure and the onset of multiple nebulous symptoms.

Over the last decade the term Breast Implant Illness has gained traction on social media platforms. In the medical literature it refers to a variety of systemic symptoms reported by women with breast implants for which there are no abnormal physical findings or laboratory evaluations to explain their symptoms.^4^ There is also no specific constellation of symptoms and no diagnostic criteria, and therefore it is functionally a diagnosis of exclusion.^5^ Previous reports have shown that some patients experience symptom improvement after implant removal.^6^ After other plausible causes for the patient’s systemic symptoms, treatment options most often include removal of the patient’s breast implants.^7^ The Breast Implant Illness social media groups and some surgeons hypothesize that not only does the implant need to be removed but also the surrounding capsular tissue “en bloc” for symptom improvement.^8^ They also believe that toxins from the implant are responsible for the symptoms are also present in the capsule, and therefore failure to entirely remove, even leaving even a small portion of the capsule during explantation, leaves these toxins behind and precludes symptom improvement. Social media posts often point to studies that suggest possible toxins such as silicone and heavy metals, and voice concerns about bacteria and fungi in or around the implants.^5^

The definition of an “en bloc” capsulectomy, as used in BII social media groups, refers to implant removal where the implant capsule remains completely intact around the implant and the implant and capsule are removed together as 1 unit.^9^ The term “en bloc” has inappropriately been overextended far from its original surgical definition.^10^ “En bloc” is incorrectly used when referring to procedures other than the treatment of malignancy where a clear margin is required for cure.^11^ Described in 1894, Halsted’s “en bloc” resection for the treatment of breast cancer included the resection of the entire breast including the pectoralis major, minor, and axillary lymph nodes in a “single swath of tissue”.^12^ In the plastic surgery literature, “en bloc” capsulectomy is defined as the complete removal of the breast implant contained within the capsule along with a contiguous layer of healthy tissue.^10^ For breast implant removal, the only scientifically proven indication for “en bloc” capsulectomy is the treatment of Breast Implant Associated Anaplastic Large Cell Lymphoma, (ALCL), a T-cell lymphoma, or other malignancies of the capsule.^2^ It can be surgically preferable to remove a ruptured gel implant while still inside the capsule to contain any gel from potentially leaking outside of the implant shell or capsule. In the case of capsular contracture, there is currently no scientifically proven benefit of removing an implant in the capsule, or removing a thin, asymptomatic capsule in a patient for a benign condition.^2^ Total capsulectomy also carries higher surgical risks, including hematoma, contour irregularities, and pneumothorax if the implant is in a subpectoral position, and often requires a larger incision and more operative time with the inherent risks involved.^2^ There are potential aesthetic complications as well, particularly when the implant lies in the sub-glandular plane under very thinned soft tissue coverage or was previously placed through a peri-areolar incision (Figure 1).

**Figure 1.**
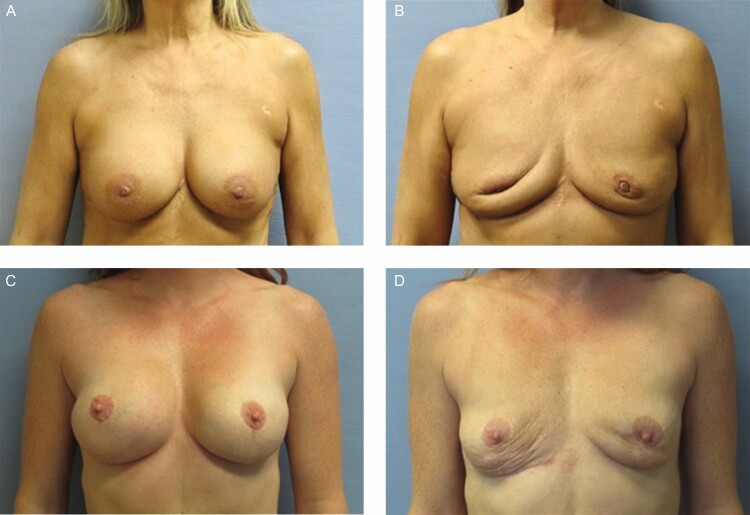
(A) A 56-year-old female patient desires explantation for self-reported BII, underwent total capsulectomy through IMF incision and the (B) resulting soft-tissue deformity with previous periareolar incision and thin capsules in subglandular placement. (C) A 45-year-old female patient desires explantation for self-reported BII, underwent intact total capsulectomy. (D) Resulting deformity with previous submuscular placement.

There currently exists a state of clinical equipoise with no consensus among the clinical community, patient advocates, or the FDA regarding the care of women who self-report BII. All concerned stakeholders have expressed the desire to collect both real world evidence of systemic symptoms as well as valid scientific evidence based on biospecimen analyses. As surgeons we are tasked with balancing the psychological and aesthetic gains of breast implant surgery against potential health risks.^13^ This study aims to reconcile the clinical equipoise and better inform clinicians, patients, and regulators. Previously published papers on symptom improvement after implant removal are retrospective, have no control cohort, or have no long term follow up.^14,15^ The purpose of this study is to address the potential etiologies that have previously been hypothesized to be associated with the development of BII.^16^ These include heavy metals, microbes, microbial toxins, cytokines, alternate causation (vitamin D deficiency, thyroid issues, heavy metals from previous tattoos or diet, and preexisting anxiety or depression or connective tissue disease [CTD]). The second goal of this study is to determine who will benefit from explantation of their breast implant and whether the type of capsulectomy – total, partial, or “en bloc” makes any difference on systemic symptom improvement and are there any baseline data points that may predict which patients may have reduced symptoms, to what extent, and for how long. The third goal of the study aims to evaluate if there is any evidence of a dose response based on number of years a patient was implanted or whether the implant fill – saline vs silicone, implant shell – texture vs smooth, or position of the device- subglandular vs submuscular has significance. Additionally, all explanted capsule tissue were photographed and analyzed to explore any potential statistical differences between the capsule pathology, microbe diversity, heavy metal content, and immunological differences between the cohorts.

## METHODS

A prospective, controlled study was designed to evaluate breast implant capsules, peripheral blood, baseline demographics, baseline patient reported systemic symptoms, and PROMIS questionnaires. The study was registered on *ClinicalTrials*.*gov* (NCT04255810) and was entirely funded by the Aesthetic Surgery Education and Research Foundation (ASERF). All patients signed an informed consent for the use of their de-identified biospecimens and surveys and the study followed the guidelines of Declaration of Helsinki and was approved by the Rhode Island Hospital Institutional Review Board (Providence, RI). The first patient was enrolled in November 2019 and the study was fully enrolled in May 2021. Patients were consecutively enrolled into 1 of 3 cohorts: (A) 50 women with implants and symptoms self-defined as BII; (B) 50 women with implants undergoing either an implant replacement or explantation without symptoms they attributed to their implants; and (C) 50 women undergoing an elective aesthetic mastopexy who had no previous exposure to any implanted device. Strict inclusion criteria required patients live within 3 hours of an investigator and have no systemic evidence of active infection or untreated malignancy and be between 30-65 years of age. Patients with any current or previous diagnosis of BIA-ALCL, HIV, breast cancer, and genetic males were excluded (Table 1). Five surgeon collaborators agreed to enroll patients, follow their enrolled subjects for a period of 1 year, and comply with a strict study protocol. The patients completed a systemic symptoms questionnaire in person, on paper at baseline, 3-6 weeks, 6-months, and 1 year post-operatively and patient surveys were de-identified and assigned a site and subject number prior to data review. This included details about allergies, menopausal status, medications, presence of dental amalgams, 22 systemic symptoms, presence of autoimmune disease, other medical issues, physicians they have seen for their symptoms, personal or family history of autoimmune disease, any significant life changes, their primary source of medical information, the type of implant (brand, fill, surface), when the implant was placed, and any previous implants. (Appendix A, available online at www.aestheticsurgeryjournal.com) Patients also completed National Institutes of Health (NIH) Patient Reported Outcomes Measurement Information System (PROMIS) questionnaires. These are a standardized set of patient-reported outcomes that cover physical, mental, and social health. PROMIS measures generate a T-score with a mean of 50 and standard deviation of 10 in a reference population (usually U.S. general population). The specific questionnaires used included Cognitive Function, Fibromyalgia, Fatigue and Anxiety/Depression. Case Report Forms (CRF) documented patient demographics including age, marital status, educational level, race, ethnicity, reproductive history, history of breast feeding and any history of mastitis, medical history, medications, tobacco or marijuana use, history of anxiety/depression, autoimmune illness, and presence of tattoos, their color, and percent body surface. Data accumulated from the completed PROMIS questionnaires and surveys were analyzed and reviewed by a clinical psychologist with expertise in anxiety.

**Table 1.** Inclusion and Exclusion Criteria

Inclusion Criteria	Exclusion criteria	
a) Genetic female	a) Has any breast disease considered to be pre-malignant in 1 or both breasts, a history of breast cancer, or an untreated cancer of any type	f) HIV positive (based on medical history)
b) Willingness to follow all study requirements including agreeing to attend all required follow up visits and signs the informed consent	b) Subject lives more than 3 hours travel from the treating surgeon	g) Has been diagnosed with BIA-ALCL
c) Agrees to donate biospecimens to the research study which may not be returned to the subjects	c) Has an abscess or infection	h) Has any medical condition such as obesity (BMI > 40), diabetes, chronic lung or severe cardiovascular disease that might result in unduly high surgical risk, and/or significant postoperative complications
d) Age 30-65	d) Is pregnant or nursing or has had a full-term pregnancy or lactated within 3 months of enrollment	i) Has been implanted with any device that has not been approved by the FDA or equivalent regulatory agency outside the US
	e) Has been implanted with any implantable medical device or silicone implant other than breast implants (except intraocular lenses)	j) Works for any breast implant manufacturer or any of their subsidiaries, the study surgeon, or are directly related to anyone that works for a breast implant manufacturer or any of their subsidiaries

**Figure 2.**
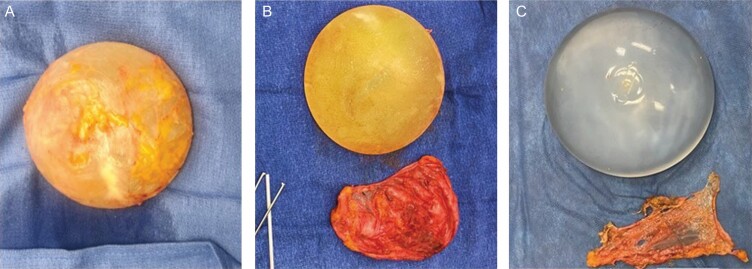
(A) A total intact capsulectomy, (B) total capsulectomy, and (C) partial capsulectomy.

Investigator surgeons completed a detailed Surgeon Observation Form on the day of surgery documenting style of implant, manufacturer, fill, shell, pocket location, description of capsule, any evidence of rupture, deflation, or double capsules, type of capsulectomy performed, and photographs of implant and capsules were required on all explants. All patients in Cohort A and B had at least a partial capsulectomy. For the purposes of this study, we divided capsulectomy into 3 types: Total intact capsulectomy- the implant was removed in the capsule with the capsule completely intact (Figure 2 and Appendix B, available online at www.aestheticsurgeryjournal.com). This is what has been described by social media as “en bloc”. Total capsulectomy- the entire capsule was removed whether with the implant in the capsule but the capsule not completely intact, or the implant was removed prior to total capsule removal, and partial capsulectomy- some capsule was left behind in the implant pocket. There were no patients who had the entire capsule left as some capsule tissue (more than a small biopsy) was removed for the multiple study specimens in all patients.

Biospecimens obtained the day of surgery included systemic blood in all 3 cohorts and capsule tissue in Cohorts A and B. Blood and capsule tissue were collected, de-identified, blinded with respect to cohort, and sent to Brown University within 24 hours of collection. Approximately 10 grams of capsule tissue was removed from each capsule, right and left sides, and sent to Eurofins Frontier Global Sciences, LLC. (Tacoma, WA), an advanced analytical laboratory specializing in heavy metals analysis. Under sterile conditions, swabs were obtained from the surface of the implant and capsule and sent along with a piece of capsular tissue to MicroGenDx (Lubbock, TX) for PCR/Next Generation Sequencing (NGS) providing a rigorous evaluation of exposure to microbes.

The remainder of the capsule tissue and blood were sent to Brown University Department of Pathology where capsule hematoxylin and eosin (H&E) histopathology was performed. Blood was tested for C-reactive Protein (CRP), thyroid levels, vitamin D level, and complete leukocyte count with differential. Within 24 hours of collection the sera were stored in 2 mL aliquots at -80C until use. One aliquot was analyzed with a BD Biosciences LSRII Analyzer/Flow Cytometer for cytokines IL-2, IL-4, IL-5, IL-6, IL-9, IL-10, IL-13, IL-17A, IL17F, IL-22, IFNγ, and tumor necrosis factor α (TNFα) with the LEGENDplex Human T**-**helper (Th) Cytokine Panel (13-plex) (Cat No 741001; BioLegend, San Diego, CA). Each analysis was controlled by an internal standard provided by the manufacturer. Prior to analysis all serum samples were diluted 1:10 to reduce background interference and analyzed in duplicate, while a subset of samples was also analyzed undiluted (neat). The remaining portion of the patient’s serum was further analyzed at the Johns Hopkins University Department of Medicine, Dermatology, Allergy, and Clinical Immunology (DACI) Reference Laboratory for antibodies specific for bacterial enterotoxin superantigens as these superantigens that have been potentially linked to a range of immune dysfunctions.^17-19^ Sera from the 3 Cohorts were analyzed by the ImmunoCAP 250 System (Thermofisher Scientific, Kalamazoo, MI, USA) for the presence and levels of IgE and IgG antibodies specific for superantigens SSA, SSB and TSST Staphylococcal enterotoxins. Antibody levels above the analytical sensitivity of the assays (IgE assay: 0.1 kUa/L where 1 IU = 2.4 ng of IgE; IgG assay; 2 mga/L where the “a” in the unit refers to “allergen-specific”) were defined as positive and an indication of enterotoxin superantigen exposure.

### Statistical Analysis

Pairs of cohorts were compared at baseline using logistic regression analyses with cohort as the dependent variable and baseline characteristic as the explanatory variable. Additionally, age and BMI were compared between pairs of cohorts using a 2-sided 2-sample *t* test. Follow-up symptom resolution at 3-6 weeks and 6 months were compared across 3 cohorts, and within Cohort A and Cohort B by method of explantation (partial, total, en bloc), using Fisher’s exact test for categorical variables and analysis of variance for percent reduction in number of symptoms.

Only patients who had the given symptom at baseline were included in the analysis. For the PROMIS data, the PROMIS Normative Score, moderate to severe, is defined as a value greater then, or equal to sixty. Comparisons were also made between pairs of cohorts using Fisher’s exact test for categorical variables and a two-sided 2 sample t-test for percent reduction in number of symptoms. A *P*-value of ≤0.05 was statistically significant.

## RESULTS

Follow-up at the time of data lock was 98%-100% at 3-6 weeks and 78%-98% at 6 months for all 3 cohorts.

### Demographics

There was no statistical difference between Cohort A, B and C with respect to age, marital status, educational level, cigarette smoking, or a history of lactation problems. The age range in Cohort A was 30-65 with a mean of 44.5, the age range in Cohort B was 30-65 with a mean of 46.9, and the age range in Cohort C was 30-63 with a mean of 46.5. There was no statistical difference in medication use except cohort A used a statistically higher amount of Aspirin/NSAIDs, prescription pain medications, and other herbal/nonprescription medicines than the other 2 cohorts. Cohort A also reported significantly more marijuana use, more tattoos, and more allergies, including allergies to medicines, pollen, mold, gluten, dust, and wheat. The BMI was higher in Cohorts A and C as compared to B. Patients were asked at baseline what was their primary source of medical information. Social media was the primary source of medical information for 58% of patients in Cohort A, 3% of patients in Cohort B, and 2% in Cohort C (Table 2 and Appendix C, available online at www.aestheticsurgeryjournal.com).

**Table 2.** Baseline Demographics

	Cohort A	Cohort B	Cohort C
Age	30-65 (mean 44.5)	30-65 (mean 46.9)	30-63 (mean 46.5)
Menopausal status	Pre-menopausal: 62% Peri-menopausal: 14% Post-menopausal: 24%	Pre-menopausal: 56% Peri-menopausal: 22% Post-menopausal: 22%	Pre-menopausal: 62% Peri-menopausal: 14% Post-menopausal: 24%
BMI	17.9-25.5 (median 26.2)	17.5-31 (median 22.4)	19-34 (median 24.9)
Implant type	Saline: 64% Gel: 36% Smooth: 90% Textured: 10%	Saline: 22% Gel: 78% Smooth: 23% Textured: 77%	
Allergies	Medicines: 48% Pollen: 50% Dust: 40% Gluten: 14%	Medicines: 36% Pollen: 52% Dust: 30% Gluten: 2%	Medicines: 28% Pollen: 26% Dust:14% Gluten: 0%
Marijuana smoker- former and current	26%	16%	14%
Education Level	High School/GED: 6% Some college/vocational school: 32% College Graduate: 42% Post-graduate education: 20%	High School/GED: 4% Some college/vocational school: 26% College Graduate: 46% Post-graduate education: 24%	High School/GED: 13% Some college/vocational school: 9% College Graduate: 55% Post-graduate education: 23%

### Medical History

There was a statistically significant increased incidence of self-reported illness in Cohort A as compared to the other 2 cohorts with respect to Fibromyalgia, Chronic Fatigue, anxiety/depression, ANA positive, Sjogren’s Syndrome, irritable bowel syndrome, rheumatological/autoimmune disease, and rheumatoid arthritis. There was no statistical difference between Cohorts A, B and C with respect to self-reported history of osteoarthritis, thyroid disease, Hashimoto’s thyroiditis, rheumatoid arthritis, presence of Rheumatoid factor, and the presence of rheumatologic/autoimmune disease or family history of rheumatologic diseases.

### Implant Characteristics

#### Cohort A

Sixty-four percent of implants in this cohort were saline and 90% had a smooth surface. Seventy-eight percent of implants were placed for primary augmentation and 22% were placed for revision augmentation. Implants were placed been 1989 and 2019 with a mean of twelve years at the time of removal. Eighty-eight percent of implants were intact, 4% had gel bleed, 4% ruptured gel, 4% deflated saline. Capsules were described as Grade 1 in 78%, Grade 2 in 8%, Grade 3 in 12%, Grade 4 in 2%. of patients. Seventy-two percent of capsules were described as thin and transparent.

#### Cohort B

Seventy-eight percent of implants in this cohort were silicone gel and 57% were textured. Seventy-four percent of implants were placed for primary augmentations and 25% were placed for revision augmentation. Implants were placed between 1990-2016 with a mean of 13.5 years. Seventy-five percent of implants were intact, 25% were ruptured silicone gel. Capsules described as Grade 1 in 43%, Grade 2 in 20%, Grade 3 in 20%, Grade 4 in 8%. Thirty-eight percent of capsules were described as thin and transparent, 33% thin and opaque, 22% thick and opaque, and 8% thick and calcified. There was no statistically significant difference in the number of years implanted, type of implant, presence of rupture or gel bleed between the cohorts.

#### Symptoms

Over 100 symptoms have been reported by women with self-described breast implant illness with no specific constellation of symptoms.^2^ The systemic symptom survey asked about the twenty-two most frequently reported symptoms and patients could write in additional symptoms. The most common symptoms reported by the Cohort A patients were fatigue (96%), brain fog (90%), joint pain (82%), muscle pain/weakness (80%), headaches (78%), low libido (78%), dry eyes (76%), memory issues (76%), anxiety (72%), and hair loss (66%), which are consistent with other reports on patients with self-reported BII. Cohorts B and C experienced significantly fewer symptoms than Cohort A with no statistically significant difference in symptoms reported between Cohort B and Cohort C, at baseline. As compared to Cohorts B and C, Cohort A reported a statistically higher incidence of headaches, low libido, abdominal pain, hair loss, dry eyes, fatigue, weight gain, memory issues, rash, heartburn, diarrhea, dry mouth, anxiety, cold intolerance, weight loss, depression, brain fog, joint pain, irregular heartbeat, insomnia, muscles pain/weakness, and numbness/tingling in extremities. The average number of baseline symptoms in Cohort A was 13.4 (range 3-22), for Cohort B 2.4 (range 0-13), and for Cohort C 1.4 (range 0-7). In Cohort B 30% reported no symptoms, the most common symptoms reported in this group were fatigue (26%) and headache (26%). Fifty percent of Cohort C reported no symptoms at baseline, the most common symptoms reported were headache (20%) and anxiety (18%). Two patients in Cohort A were diagnosed with other diseases during the study follow up period, 1 patient who did not achieve symptom relief was diagnosed with a brain tumor 1 year post operatively, the second patient was diagnosed with multiple myeloma 6 months post operatively and is undergoing treatment.

### PROMIS Data

**Figure 3.**
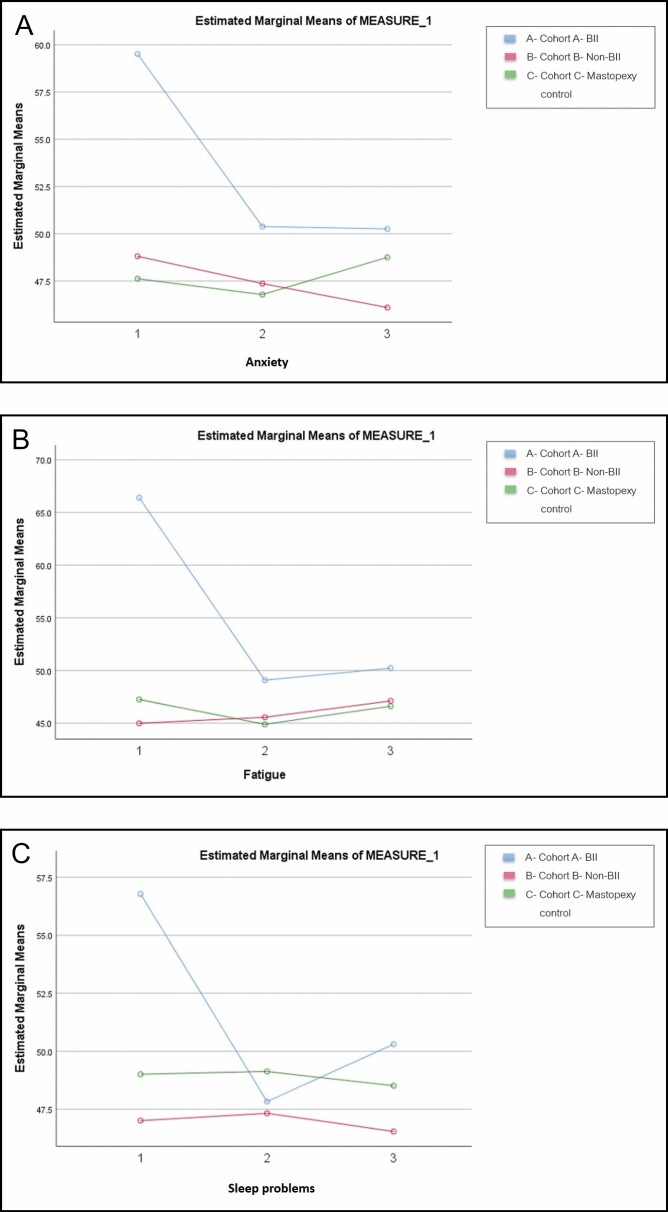
PROMIS Score Change Over Time. (A) PROMIS score demonstrates change over time for Anxiety at Baseline (1), 3-6 weeks (2), and 6-months (3). (B) PROMIS score demonstrates change over time for Fatigue at Baseline (1), 3-6 weeks (2), and 6-months (3). (C) PROMIS score demonstrates change over time for Sleep Disturbance at Baseline (1), 3-6 weeks (2), and 6-months (3) Sleep disturbances.

The PROMIS baseline data showed a statistically higher level of fatigue, anxiety, and sleep disturbance in Cohort A as compared to the other 2 cohorts. Cohort A’s anxiety, fatigue, and sleep disturbance improve from baseline to 3-6 weeks and their scores for anxiety and fatigue were stable through 6-month follow-up but remained higher at all time points than the other 2 cohorts. At 3-6 weeks, 78% of Cohort A approached normalized scores for the PROMIS anxiety value (from the severe or moderate range to mild or none) which increased to 86% of patients in Cohort A demonstrating the same at 6 months. Fatigue normalized in 85% of Cohort A at 3-6 weeks and was normalized in 79% of the Cohort at 6 months. Eighty-three percent of Cohort A had normalized sleep disturbance at 3-6 weeks to 73% at 6 months. Most patients with self-reported BII showed normalized fatigue and anxiety levels after explant by 3-6 weeks post-surgery which appeared stable to 6 months. Between 20-25% of patients in Cohort A did not improve. There was no statistically significant difference in normalization of the PROMIS data between the capsulectomy types. All patients in Cohort A showed normalization toward mild to moderate in all 3 parameters measured, however their scores were consistently higher than the other 2 cohorts. (**Figure 3**)

### Symptom Improvement

#### Cohort A (BII Cohort)

Ninety-four percent of the patients in Cohort A had at least partial improvement in the number of symptoms reported at baseline, with a reduction of 2-22 symptoms reported at the 6 month follow up. Five patients had complete resolution of their symptoms, 2 of those patients had a total capsulectomy and 2 had a partial capsulectomy and 1 had an intact total capsulectomy. Three patients had no symptom improvement at 6 months, all 3 had a total capsulectomy. At 3-6 weeks patients in Cohort A had a 55% reduction in the number of symptoms reported, at 6 months there was a 68% reduction in the number of symptoms with 98% follow-up. All reported baseline symptoms showed significant improvement at 3-6 weeks and further improvement was seen at 6 months. Fatigue, brain fog, joint pain, and muscle pain and weakness showed the greatest reduction in reporting by Cohort A, fatigue decreased from 96% to 34% of subjects, brain fog from 90% to 22%, joint pain from 82% to 25%, and muscle pain and weakness from 80% to 22% of subjects. The type of capsulectomy, intact total, total, or partial, showed similar symptom improvement with no statistically significant difference in the reduction in the number of symptoms based on the type of capsulectomy performed.

#### Cohort B (Non-BII Cohort)

Thirty percent of Group B reported no symptoms at baseline, which increased to 34% of subjects reporting no symptoms at 6 months with 98% follow up. Group B had improvement between baseline and 6 month follow up for headaches from 26%-18% of patients reporting, heartburn reduced from 10% to 4.5%, muscle pain and weakness 10%-4%, fatigue 26%-15%, join pain 16%-9%, and insomnia 12%-4.5%. Group B patients did, however, report and increase in the percentage of rashes from 4%-9%, and memory issues increased from 4% of patients reporting at baseline to 7% at 6 months.

#### Cohort C (Control Cohort)

**Figure 4.**
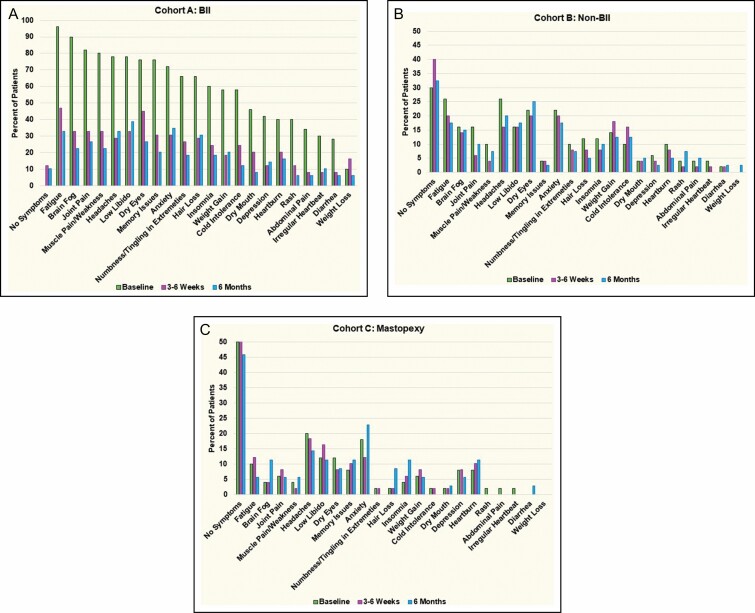
(A-C) Comparison of symptom resolution between the cohorts at 3-6 weeks and 6 months.

Fifty percent of subjects in Cohort C reported no systemic symptoms at baseline, this number decreased to 44% of subjects reporting no symptoms. At the 6 month follow up visit cohort C reported a decrease in headaches from 20% of subjects to 14%, dry eyes 12%-9%, and fatigue 10%-6% with 78% follow-up. However, Cohort C self-reported an increase in anxiety from 18% of subjects to 23%, hair loss 2%-9%, and memory issues 8%-12%, brain fog 4%-11%, and insomnia 4%-11%. (Figure 4)

## Discussion

There is extensive literature on a possible connection between breast implants and systemic symptoms or defined connective tissue disease. Included in the existing literature are women with both saline and silicone implants and studies that fail to apply any conventional diagnostic criteria. Most studies lack identification of the type of implant, offer unknown disease status prior to implantation, lack a consistent latent period or information on length of exposure, provide inconsistent documentation of effects on symptoms after explantation, and consistently lack long-term follow-up.^20^ The difficulty in defining BII is that most self-reported signs and symptoms are commonly found in the general population.^21^ The constellation of symptoms that characterize BII are also found in multiple other illnesses, such as chronic fatigue syndrome, fibromyalgia, and multiple chemical sensitivities. In this study, data from both a self-reported systemic symptoms questionnaire and validated NIH PROMIS questionnaire were collected before and after surgery at defined post-operative intervals to provide valid and reliable Patient Reported Outcomes Measures (PROM) of health concepts relevant to clinicians and researchers.^22^

This part 1 of the ASERF Systemic Symptoms in Women Biospecimen Analysis Study focuses on the impact of capsulectomy type and systemic symptom improvement. This study enrolled 150 patients and followed strict inclusion and exclusion criteria. Data was prospectively collected from sequential patients at 5 investigator sites within the United States. Follow-up was robust, with 98%-100% follow-up at 3-6 weeks for all 3 cohorts, 78%-98% at 6-months, and 1 year data is currently at 80%. The baseline symptom surveys showed a statistically significant difference in self-reported symptoms between Cohort A and the 2 control cohorts B and C. There was no statistically significant difference in symptoms between Cohort B and C consistent with the findings of Misere, et al suggesting a potential bias among self-designated breast implant illness patients.^23^ This study demonstrated at least partial symptom improvement in 96% of patients in Cohort A, with no specific constellation of symptoms or history that predicted this improvement except for self-reporting BII and undergoing an explantation. The type of capsulectomy was recorded by the surgeon and further documented by photography. The results demonstrated that intact total, total, or partial capsulectomies all showed similar symptom improvement with no statistical difference in the reduction of symptoms based on the type of capsulectomy (Appendix D, available online at www.aestheticsurgeryjournal.com). There was also no difference in the dose response based on the number of years implanted as the average and mean number of years implanted did not vary between the cohorts. There was a statistically significant difference between implant shell surface and fill with more smooth saline implants identified in Cohort A. There was no statistical significance in the presence gel rupture or bleed between the cohorts.

The limitations of this study include the relatively small cohort size which was in part based on the expense of the biospecimen analysis. These analyses should be considered exploratory as many comparisons were made and it is expected that some results are statistically significant due to chance alone. However, the size of each cohort was determined to be large enough to provide statistical significance to the symptoms data collected. Further, there are inherent limitations to patient reported symptoms and medical histories. All patients had at least a partial capsulectomy as capsules were sent for study analysis on all subjects in the implant cohorts.

In addition to the qualitative systemic symptom data analyzed in this study, biospecimen data was collected from all cohorts. This included the analysis of capsule tissue for the presence of twenty-two heavy metals, microbes within the capsule and on the implant surface, the evaluation of patient’s blood for the presence of any antibodies to microbial toxin/superantigens and elevated serum cytokine levels, and possible alternate causations such as vitamin D deficiency or thyroid disease. Data was also collected to document potential sources of elevated heavy metals such tattoos or diet. This data will be published in Part 2 and 3 of the ASERF study.

## Conclusions

This study demonstrated at least partial symptom improvement in patients with self-reported systemic symptoms following removal of their implants. After excluding other potential causes for their symptoms, implant removal can be discussed with patients who believe that their implants are responsible for their symptoms. The data indicates that women who self-report BII and have elevated anxiety and experience improvement in their anxiety after explantation which is sustained at least to 6 months. The data, however, does not support the requirement for performing “en bloc” removal of the breast implant and capsule in women self-reporting BII. Further, “en bloc” is a surgical procedure that is indicated only for oncologic surgery. We report subjects from Cohort A who had a complete symptom resolution persisting through 6 months without a total capsulectomy, demonstrating that symptom improvement is independent of the presence of capsule remaining in the pocket. Total intact capsulectomy, removing the entire implant and capsule together as 1 unit may require a longer incision and carries higher risks than a total, partial, or no capsulectomy. Patients considering implant removal for systemic symptoms that they attribute to their implants should weigh the risks and benefits of explantation. Physicians and their patients should make informed decisions based on scientifically obtained data rather than the recommendations of social media.

## Supplementary Material

sjab417_suppl_Supplementary_Appendix_A

sjab417_suppl_Supplementary_Appendix_B

sjab417_suppl_Supplementary_Appendix_C

sjab417_suppl_Supplementary_Appendix_D
