# Supplementary material for: Impact of Capsulectomy Type on Post-Explantation Systemic Symptom Improvement: Findings From the ASERF Systemic Symptoms in Women-Biospecimen Analysis Study: Part 1
Source: Aesthet Surg J. 2021 Dec 16;42(7):809–19. doi: 10.1093/asj/sjab417 (PMC9208825; doi:10.1093/asj/sjab417)
Supplement: sjab417_suppl_Supplementary_Appendix_A [file sjab417_suppl_supplementary_appendix_a.docx]

**Appendix A**. ASERF Study: Analysis of Baseline Demographic and Systemic Symptoms Between the Cohorts

|  | COHORT A vs. COHORT B | COHORT A to COHORT C | | | |
| --- | --- | --- | --- | --- | --- |
| Baseline Characteristic | Reference Category | Odds Ratio | p-value | Odds Ratio | p-value |
| Age (years) (continuous) | - | 0.968 | 0.1712 | 0.974 | 0.2501 |
| Marital Status | Single |  |  |  |  |
| Married |  | 2.868 | 0.0979 | 0.951 | 0.9462 |
| Widowed |  | 1.250 | 0.8697 | >999.999 | 0.2588 |
| Separated |  | - | - | - | - |
| Divorced |  | 3.750 | 0.1312 | 1.200 | 0.8447 |
| BMI (kg/m^2) (continuous) | - | 1.288 | **0.0002** | 1.044 | 0.3701 |
| Lactation Problems History (Yes/No) | No | 0.667 | 0.4731 | 0.952 | 0.9297 |
| Tobacco History | Never |  |  |  |  |
| Former |  | 1.304 | 0.5634 | 2.529 | 0.0725 |
| Current |  | >999.999 | 0.0864 | >999.999 | 0.0734 |
| Marijuana Use | Never |  |  |  |  |
| Former |  | 1.277 | 0.6479 | 3.486 | 0.0758 |
| Current |  | >999.999 | **0.0156** | 1.163 | 0.8390 |
| Medications |  |  |  |  |  |
| Antibiotics (Yes/No) | No | 2.538 | 0.1096 | 13.820 | **0.0138** |
| Antidepressants (Yes/No) | No | 2.632 | 0.0583 | 1.102 | 0.8257 |
| Anti-anxiety Medications (Yes/No) | No | 2.068 | 0.1889 | 1.285 | 0.6176 |
| Antihypertensive Medication (Yes/No) | No | 2.667 | 0.2552 | 1.278 | 0.7273 |
| Aspirin/NSAID (Yes/No) | No | 14.461 | **<0.0001** | 10.615 | **<0.0001** |
| Birth Control (Yes/No) | No | 0.574 | 0.4655 | 0.734 | 0.6960 |
| Hormonal Replacement Therapy (HRT) (Yes/No) | No | 2.875 | 0.0936 | 2.875 | 0.0936 |
| Prescription Pain Medications (Yes/No) | No | >999.999 | **0.0032** | 1.568 | 0.5076 |
| Thyroid Medication (Yes/No) | No | 1.285 | 0.6176 | 1.733 | 0.3012 |
| Other-Herbal-non-prescription medicines (Yes/No) | No | 4.636 | **0.0060** | 1.325 | 0.5171 |

Note: COHORT A (BII) Those patients seeking explantation because they believe their implants are responsible for their symptoms. COHORT B (Non-BII) Patients requesting implant exchange or explantation without self-reported ‘BII’; Cohort C Patients undergoing mastopexy, without soft tissue support or any previous implants. The odds ratios and p-values are from a logistic regression analysis with group as the dependent variable and the baseline characteristic as the explanatory variable. The p-value is for a two-sided test of the null hypothesis that the true odds ratio equals 1. PROMIS Normative Score: Moderate to severe defined as a value >=60.

|  | COHORT A vs. COHORT B | COHORT A to COHORT C | | | |
| --- | --- | --- | --- | --- | --- |
| Baseline Characteristic | Reference Category | Odds Ratio | p-value | Odds Ratio | p-value |
| Osteoarthritis (Yes/No) | No | 3.128 | 0.3308 | 3.128 | 0.3308 |
| Thyroid Disease: Hypothyroid (self-report) (Yes/No) | No | 1.285 | 0.6176 | 3.173 | 0.0640 |
| Thyroid Disease: Hashimoto’s (self-report) (Yes/No) | No | 1.362 | 0.6960 | 2.043 | 0.4223 |
| Rheumatological or Autoimmune Disease (Yes/No) | No | 11.345 | **0.0002** | >999.999 | **<0.0001** |
| Rheumatoid Arthritis (Yes/No) | No | >999.999 | **0.0392** | >999.999 | **0.0392** |
| Sjogren’s Syndrome (Yes/No) | No | >999.999 | **0.0169** | >999.999 | **0.0169** |
| ANA Positive (Yes/No) | No | >999.999 | **0.0073** | >999.999 | **0.0073** |
| Rheumatoid Factor-positive (Yes/No) | No | >999.999 | 0.0935 | >999.999 | 0.0935 |
| Fibromyalgia (Yes/No) | No | >999.999 | **0.0392** | >999.999 | **0.0392** |
| Chronic Fatigue Syndrome (Yes/No) | No | >999.999 | **0.0073** | >999.999 | **0.0073** |
| Irritable Bowel Syndrome (Yes/No) | No | 6.767 | **0.0166** | 6.626 | **0.0179** |
| History of Anxiety or Depression (Yes/No) | No | 3.222 | **0.0055** | 2.071 | 0.0734 |
| Symptoms |  |  |  |  |  |
| Headaches (Yes/No) | No | 10.091 | **<0.0001** | 13.827 | **<0.0001** |
| Low Libido (Yes/No) | No | 18.611 | **<0.0001** | 25.409 | **<0.0001** |
| Abdominal Pain (Yes/No) | No | 12.364 | **0.0013** | 24.727 | **0.0023** |
| Hair Loss (Yes/No) | No | 14.231 | **<0.0001** | 93.165 | **<0.0001** |
| Dry Eyes (Yes/No) | No | 11.227 | **<0.0001** | 22.694 | **<0.0001** |
| Fatigue (Yes/No) | No | 68.308 | **<0.0001** | 211.199 | **<0.0001** |
| Weight Gain (Yes/No) | No | 8.483 | **<0.0001** | 21.174 | **<0.0001** |
| Memory Issues (Yes/No) | No | 76.000 | **<0.0001** | 35.624 | **<0.0001** |
| Rash (Yes/No) | No | 10.444 | **0.0004** | 32.000 | **0.0010** |
| Heartburn (Yes/No) | No | 6.000 | **0.0012** | 7.499 | **0.0007** |
| Diarrhea (Yes/No) | No | 19.050 | **0.0053** | >999.999 | **<0.0001** |
| Dry Mouth (Yes/No) | No | 20.443 | **<0.0001** | 40.889 | **0.0004** |

|  | COHORT A vs. COHORT B | COHORT A to COHORT C | | | |
| --- | --- | --- | --- | --- | --- |
| Baseline Characteristic | Reference Category | Odds Ratio | p-value | Odds Ratio | p-value |
| Anxiety (Yes/No) | No | 9.117 | **<0.0001** | 11.428 | **<0.0001** |
| Cold Intolerance (Yes/No) | No | 12.429 | **<0.0001** | 66.283 | **<0.0001** |
| Weight Loss (Yes/No) | No | >999.999 | **0.0073** | >999.999 | **0.0077** |
| Depression (Yes/No) | No | 11.345 | **0.0002** | 8.145 | **0.0004** |
| Brain Fog (Yes/No) | No | 47.250 | **<0.0001** | 211.499 | **<0.0001** |
| Joint Pain (Yes/No) | No | 23.910 | **<0.0001** | 69.836 | **<0.0001** |
| Irregular Heartbeat (Yes/No) | No | 10.286 | **0.0030** | 20.563 | **0.0042** |
| Insomnia (Yes/No) | No | 10.998 | **<0.0001** | 35.236 | **<0.0001** |
| Muscle pain/weakness (Yes/No) | No | 36.000 | **<0.0001** | 94.000 | **<0.0001** |
| Numbness/tingling in extremities (Yes/No) | No | 17.471 | **<0.0001** | 93.165 | **<0.0001** |
| Fam Hx Auto- immune or Connective Tissue Dis (Yes/No) | No | 1.025 | 0.9622 | 1.612 | 0.4027 |
| Experienced a Significant Personal Loss(es) in the Past Year (Yes/No) | No | 0.755 | 0.5171 | 1.517 | 0.3797 |
| Any Allergy (Yes/No) | No | 1.405 | 0.4769 | 4.909 | **0.0005** |
| Allergy to Medicine(s) (Yes/No) | No | 1.641 | **0.2254** | 2.308 | **0.0488** |
| Allergy: Pollen (Yes/No) | No | 0.923 | 0.8415 | 2.769 | **0.0178** |
| Allergy: Mold (Yes/No) | No | 1.098 | 0.8289 | 3.373 | **0.0220** |
| Allergy: Gluten (Yes/No) | No | 7.977 | 0.0566 | >999.999 | **0.0015** |
| Allergy: Dust (Yes/No) | No | 1.714 | 0.2071 | 4.000 | **0.0056** |
| Allergy: Milk/Dairy (Yes/No) | No | 3.907 | 0.1001 | 7.814 | 0.0592 |
| Allergy: Soy (Yes/No) | No | >999.999 | 0.2373 | >999.999 | 0.2408 |
| Allergy: Tree Nuts (Yes/No) | No | 1.000 | 1.0000 | 3.064 | 0.3397 |
| Allergy: Wheat (Yes/No) | No | 4.261 | 0.2023 | >999.999 | **0.0177** |
| Allergy: Eggs (Yes/No) | No | <0.001 | 0.2373 | - | - |
| Allergy: Shellfish/Fish (Yes/No) | No | 1.000 | 1.0000 | 0.480 | 0.5540 |
| Allergy: Peanuts (Yes/No) | No | >999.999 | 0.2373 | 0.980 | 0.9885 |

|  | COHORT A vs. COHORT B | COHORT A to COHORT C | | | |
| --- | --- | --- | --- | --- | --- |
| Baseline Characteristic | Reference Category | Odds Ratio | p-value | Odds Ratio | p-value |
| Breast Implant: Saline (Yes/No) | No | 4.750 | **0.0004** | - | - |
| Breast Implant: Silicone Gel (Yes/No) | No | 0.211 | **0.0004** | - | - |
| Breast Implant: Smooth (Yes/No) | No | 9.332 | **<0.0001** | - | - |
| Breast Implant: Textured (Yes/No) | No | 0.107 | **<0.0001** | - | - |
| Revision of Breast Implants (Yes/No) | No | 1.631 | 0.2724 | - | - |
| Capsular Contracture History (Yes/No) | No | 0.432 | 0.1247 | - | - |
| Rupture History (Yes/No) | No | 1.536 | 0.4266 | - | - |
| PROMIS Normative Score: |  |  |  |  |  |
| Fatigue (moderate to severe) (Yes/No) | No | 38.500 | **<0.0001** | 47.250 | **<0.0001** |
| Anxiety (moderate to severe) (Yes/No) | No | 2.923 | **0.0138** | 10.615 | **<0.0001** |
| Sleep (moderate to severe) (Yes/No) | No | 5.062 | **0.0035** | 8.812 | **0.0011** |
| Tattoos (Yes/No) | No | 2.681 | **0.0172** | 1.621 | 0.2309 |
| Percent Body Surface Area (continuous) | - | 1.037 | 0.2883 | 1.043 | 0.1783 |
| Green Tattoos (Yes/No) | No | 2.333 | 0.1851 | 1.400 | 0.5772 |
| Low 25-OH Vitamin D Test Results (Yes/No) | No | 0.600 | 0.2184 | 0.979 | 0.9593 |
| Hypothyroid (Yes/No) | No | 3.286 | 0.1587 | 0.651 | 0.4518 |
| CRP Elevation (Yes/No) | No | 3.632 | 0.0661 | 5.684 | **0.0322** |
| Dental Amalgams (Yes/No) | No | 1.862 | 0.1443 | 2.233 | 0.0671 |
| Thin Capsule (Yes/No) | No | 2.890 | **0.0367** | - | - |
| Thick Capsule (Yes/No) | No | 0.346 | **0.0367** | - | - |
| Baker Grade Contracture (Yes/No) | No | 0.542 | 0.2233 | - | - |
| Implant Rupture (Yes/No) | No | 0.388 | 0.0806 | - | - |
