# Supplementary material for: Impact of Capsulectomy Type on Post-Explantation Systemic Symptom Improvement: Findings From the ASERF Systemic Symptoms in Women-Biospecimen Analysis Study: Part 1
Source: Aesthet Surg J. 2021 Dec 16;42(7):809–19. doi: 10.1093/asj/sjab417 (PMC9208825; doi:10.1093/asj/sjab417)
Supplement: sjab417_suppl_Supplementary_Appendix_B [file sjab417_suppl_supplementary_appendix_b.docx]

**Appendix B**. Systemic Symptoms Questionnaire

| 1. Do you have allergies to any of the following? | 2. Do you have any of the following  symptoms? | | 3. Have you been diagnosed with any of the following? |
| --- | --- | --- | --- |
| Medicines: __________  Pollen  Mold  Gluten  Dust  Milk/Dairy  Soy  Wheat  Eggs  Shellfish/Fish  Tree nuts | Headache  Dry Eyes  Heartburn  Anxiety  Muscle Pain/weakness  Low libido  Fatigue  Diarrhea  Weight loss  Abdominal pain  Cold intolerance | Depression  Numbness/tingling  Hair loss  Memory issues  Brain fog  Irregular heartbeat  Dry mouth  Joint pain  Insomnia  Hair loss  Rash | Fibromyalgia  Endocrine dysfunction  Hypothyroidism  Hashimoto's Thyroiditis  Graves' disease  Lyme disease  Irritable Bowel Disease  Inflammatory Bowel disease  Vitamin D deficiency |
| 4. Have you seen other physicians regarding your symptoms? | 5. Family history of autoimmune disease? | | 6. Have you experienced any of the following personal losses in the last year? |
| Primary Care  Infectious disease  Rheumatologist  Neurologist  Integrative Wellness  Other? |  | | None  Death of a loved one  Son or daughter moved far away  Job loss  Loss of reputation or status  Loss of financial security  Other _______________ |
| 7. Do you have dental Amalgam fillings? | 8. Do you have a history of a medical conditions unrelated to symptoms listed? | | 9. Are you taking HRT? |
| 10. List prescribed medications last 3 months | 11. Are you taking Over the Counter supplements? | | 12. Menopausal status? |
