# Supplementary material for: Impact of Capsulectomy Type on Post-Explantation Systemic Symptom Improvement: Findings From the ASERF Systemic Symptoms in Women-Biospecimen Analysis Study: Part 1
Source: Aesthet Surg J. 2021 Dec 16;42(7):809–19. doi: 10.1093/asj/sjab417 (PMC9208825; doi:10.1093/asj/sjab417)
Supplement: sjab417_suppl_Supplementary_Appendix_C [file sjab417_suppl_supplementary_appendix_c.docx]

**Appendix C**. Defining Capsulectomy and Indications

| Year | Type of Capsulectomy | Indication | Contraindication | Author |
| --- | --- | --- | --- | --- |
| 1993 | Total capsulectomy | Subglandular implants with capsular contracture, rupture, extracapsular silicone, calcified capsule | Submuscular only with compelling justification | Spear SL^1^ |
|  | Partial capsulectomy | Subglandular implants to reduce palpability or problems related to seroma |  |  |
| 1998 | Total capsulectomy | No replacement, pocket change, subglandular capsular contracture, calcified capsule, ruptured gel, infection | Extracapsular gel with extension to axilla due to risk of brachial plexus or vascular injury, submuscular capsules densely adherent to ribs and intercostal muscles | Young L^2^ |
|  | Partial capsulectomy | Submuscular capsule for portions that can be safely removed |  |  |
| 2012 | Total capsulectomy | Capsular contracture, rupture | Low BMI, atrophy of the chest wall, submuscular position, previous use of tissue expanders, absence of normal pectoral musculature | Gascoigne A Malata C^3^ |
|  | Partial capsulectomy | Submuscular placement |  |  |
|  | En bloc | Malignancy |  |  |
| 2018 | Total capsulectomy | Capsular contracture, calcified or thick capsules, removal silicone granulomas, benign late seroma  no replacement, pocket change | Removal of thin capsule that may cause damage to surrounding tissues, submuscular capsule densely adherent to ribs and intercostal muscles, subglandular thin patient | Florin W, Haiavy, J^4^ |
|  | En bloc | BIA-ALCL- oncologic procedure | Patients without a diagnosis of BIA-ALCL |  |
|  | Partial capsulectomy or no capsulectomy | Downsizing, patient request, capsular contracture (uncomplicated), asymptomatic textured implant with normal capsule |  |  |
| 2019 | Precise total or partial capsulectomy | Removal with or without replacement for anxiety related to texture in asymptomatic patient | Unable to safely remove capsule and patient warned of potential risks | McGuire P, Deva A, Glicksman C, et al^5^ |
|  | En bloc | Late Seroma or mass after recommended work-up and diagnosis of BIA-ALCL |  |  |
| 2021 | En bloc | Confirmed malignancy, BIA-ALCL |  | Tamma N et al^6^ |
|  | Total capsulectomy | Capsular contracture, rupture, thickened capsule, patient preference | Submuscular placement |  |
|  | Partial or anterior capsulectomy | BII, aesthetic deformities, capsular contracture, patient preference, implant rupture |  |  |
| 2021 | En bloc | BIA-ALCL stage 1A-1C |  | Calobrace B^7^ |
|  | Total capsulectomy | Capsular contracture, calcified capsule, silicone rupture, thickened fibrous capsules | Risk of rib injury or pneumothorax submuscular capsules, risk of loss of soft tissue support, implant coverage |  |
|  | Partial capsulectomy or no capsulectomy | Implant malposition, pocket change | Pathologic capsule or late seroma |  |

**References for Table 3**

^1^Spear SL, *Plast Reconstr Surg*. 1993;92(2): 323-324. PMID: 8337283.

^2^ Young L *Plast Reconstr Surg.* 102(3): 884-891.

^4^ Gascoigne A, Malata, C, Pleural Damage During Capsulectomy and Exchange of Long-standing Breast Implants in

Poland Syndrome, *Annals of Plastic Surgery,* Vol.69, Number 2, August 2012, doi:10.1097/sap.0b013e318226b4c4.

^5^ Florin W., Haiavy J. Capsular Contracture in Breast Augmentation: Medical Management and Indications for

Capsulectomy. *American Journal of Cosmetic Surgery,* 35(3), 110–123, 2018, doi:10.1177/0748806818761718.

^6^ McGuire P, Deva A, Glicksman C, et al. *Aesthetic Surg. Journal Open Forum*, 1-3, 2019, DOI: 10.1093/asjof/ojz025

www.asjopenforum.com.

*^7^* Tamma N, et al., Strategies in Breast Explantation Surgery, *Plast Reconstr Surg*. Vol.147, Number 4, 2021*,*

https://doi.org/10.1097/prs.0000000000007784.

*^8^* Calobrace MB*,* Management of Patients with Textured Implants, *Plast Reconstr Surg*. Vol.147, Number 5S,

2021, https://doi.org/10.1097/prs.0000000000008041.
