# Supplementary material for: Impact of Capsulectomy Type on Post-Explantation Systemic Symptom Improvement: Findings From the ASERF Systemic Symptoms in Women-Biospecimen Analysis Study: Part 1
Source: Aesthet Surg J. 2021 Dec 16;42(7):809–19. doi: 10.1093/asj/sjab417 (PMC9208825; doi:10.1093/asj/sjab417)
Supplement: sjab417_suppl_Supplementary_Appendix_D [file sjab417_suppl_supplementary_appendix_d.docx]

**Appendix D**. Impact of Method of Explantation (Partial, Total, Or Total Intact) on the Resolution Of Symptoms At 3-6 Weeks And 6 Months

| COHORT A: BII Patients | | | | | | |  |
| --- | --- | --- | --- | --- | --- | --- | --- |
| Dependent Variable | Time Point | Statistic | Partial | Total | Intact Total | p-value (1) | |
|  | | | | | | | |
| Resolution of Individual Symptoms: |  |  |  |  |  |  | |
|  | | | | | | | |
| Fatigue - not present (2) | 3-6 Weeks | n (%) | 3 ( 50.0) | 15 ( 57.7) | 6 ( 42.9) | 0.7690 | |
|  | 6 Months | n (%) | 5 ( 83.3) | 17 ( 63.0) | 9 ( 64.3) | 0.8221 | |
| Anxiety - not present (2) | 3-6 Weeks | n (%) | 2 ( 40.0) | 14 ( 73.7) | 5 ( 50.0) | 0.2929 | |
|  | 6 Months | n (%) | 3 ( 60.0) | 11 ( 55.0) | 6 ( 60.0) | 1.0000 | |
| Brain Fog - not present (2) | 3-6 Weeks | n (%) | 2 ( 50.0) | 16 ( 66.7) | 9 ( 60.0) | 0.7236 | |
|  | 6 Months | n (%) | 3 ( 75.0) | 19 ( 76.0) | 11 ( 73.3) | 1.0000 | |
| Muscle Pain - not present (2) | 3-6 Weeks | n (%) | 3 (100.0) | 14 ( 63.6) | 9 ( 64.3) | 0.6729 | |
|  | 6 Months | n (%) | 2 ( 66.7) | 16 ( 72.7) | 11 ( 78.6) | 1.0000 | |
| Joint Pain - not present (2) | 3-6 Weeks | n (%) | 2 ( 40.0) | 12 ( 60.0) | 10 ( 71.4) | 0.4306 | |
|  | 6 Months | n (%) | 3 ( 60.0) | 15 ( 71.4) | 9 ( 64.3) | 0.8065 | |
| Headache - not present (2) | 3-6 Weeks | n (%) | 3 (100.0) | 13 ( 56.5) | 7 ( 63.6) | 0.5178 | |
|  | 6 Months | n (%) | 3 (100.0) | 12 ( 50.0) | 8 ( 72.7) | 0.2175 | |
| Dry Eyes - not present (2) | 3-6 Weeks | n (%) | 2 ( 50.0) | 10 ( 45.5) | 5 ( 45.5) | 1.0000 | |
|  | 6 Months | n (%) | 3 ( 75.0) | 16 ( 72.7) | 7 ( 63.6) | 0.8684 | |

| COHORT A: BII Patients | | | | | | |
| --- | --- | --- | --- | --- | --- | --- |
| Dependent Variable | Time Point | Statistic | Partial | Total | Intact Total | p-value (1) |
|  |  |  |  |  |  |  |
| Percent Reduction in Number of Symptoms | 3-6 Weeks | Mean (SD) | 62.4 (44.03) | 52.7 (43.41) | 58.5 (32.61) | 0.8273 |
|  | 6 Months | Mean (SD) | 76.9 (27.64) | 62.9 (32.09) | 66.0 (29.34) | 0.6056 |
| 50% or More Reduction in Symptom Number | 3-6 Weeks | n (%) | 4 ( 66.7) | 17 ( 63.0) | 10 ( 66.7) | 1.0000 |
|  | 6 Months | n (%) | 5 ( 83.3) | 19 ( 67.9) | 12 ( 80.0) | 0.6487 |
| 80% or More Reduction in Symptom Number | 3-6 Weeks | n (%) | 2 ( 33.3) | 10 ( 37.0) | 5 ( 33.3) | 1.0000 |
|  | 6 Months | n (%) | 4 ( 66.7) | 11 ( 39.3) | 8 ( 53.3) | 0.3919 |
| Resolution of Severity of Symptoms: PROMIS^®^ |  |  |  |  |  |  |
| PROMIS^®^ normalized anxiety value (3) | 3-6 Weeks | n (%) | 1 (100.0) | 12 ( 80.0) | 5 ( 71.4) | 1.0000 |
|  | 6 Months | n (%) | 1 (100.0) | 13 ( 86.7) | 4 ( 80.0) | 1.0000 |
| PROMIS^®^ normalized fatigue value (3) | 3-6 Weeks | n (%) | 2 ( 66.7) | 22 ( 91.7) | 11 ( 78.6) | 0.2666 |
|  | 6 Months | n (%) | 1 ( 50.0) | 19 ( 79.2) | 10 ( 83.3) | 0.6271 |
| PROMIS^®^ normalized sleep value (3) | 3-6 Weeks | n (%) | 2 (100.0) | 7 ( 87.5) | 6 ( 75.0) | 1.0000 |
|  | 6 Months | n (%) | 1 (100.0) | 6 ( 75.0) | 4 ( 66.7) | 1.0000 |

| COHORT B: Non-BII Patients | | | | | | |
| --- | --- | --- | --- | --- | --- | --- |
| Dependent Variable | Time Point | Statistic | Partial | Total | Intact Total | p-value (1) |
|  | | | | | | |
| Resolution of Individual Symptoms: |  |  |  |  |  |  |
| Fatigue - not present (2) | 3-6 Weeks | n (%) | 3 ( 60.0) | 2 ( 40.0) | 0 | 0.3007 |
|  | 6 Months | n (%) | 3 ( 75.0) | 4 ( 80.0) | 1 ( 33.3) | 0.5758 |
| Anxiety - not present (2) | 3-6 Weeks | n (%) | 4 (100.0) | 2 ( 33.3) | 0 | 0.0606 |
|  | 6 Months | n (%) | 2 ( 50.0) | 2 ( 40.0) | 0 | 1.0000 |
| Brain Fog - not present (2) | 3-6 Weeks | n (%) | 2 ( 50.0) | 1 ( 33.3) | 0 | 1.0000 |
|  | 6 Months | n (%) | 2 ( 50.0) | 1 ( 33.3) | 1 (100.0) | 1.0000 |
| Muscle Pain - not present (2) | 3-6 Weeks | n (%) | 2 (100.0) | 1 (100.0) | 2 (100.0) | - |
|  | 6 Months | n (%) | 1 ( 50.0) | 1 (100.0) | 2 (100.0) | 1.0000 |
| Joint Pain - not present (2) | 3-6 Weeks | n (%) | 2 ( 50.0) | 3 (100.0) | 1 (100.0) | 0.5714 |
|  | 6 Months | n (%) | 3 ( 75.0) | 3 (100.0) | 1 (100.0) | 1.0000 |
| Headache - not present (2) | 3-6 Weeks | n (%) | 2 ( 40.0) | 3 ( 50.0) | 0 | 0.7669 |
|  | 6 Months | n (%) | 1 ( 33.3) | 4 ( 80.0) | 1 ( 50.0) | 0.7143 |
| Dry Eyes - not present (2) | 3-6 Weeks | n (%) | 1 ( 33.3) | 4 ( 57.1) | 0 | 1.0000 |
|  | 6 Months | n (%) | 0 | 3 ( 50.0) | 0 | 0.6250 |

| COHORT B: Non-BII Patients | | | | | | |
| --- | --- | --- | --- | --- | --- | --- |
| Dependent Variable | Time Point | Statistic | Partial | Total | Intact Total | p-value (1) |
|  | | | | | | |
| Percent Reduction in Number of Symptoms | 3-6 Weeks | Mean (SD) | 47.4 (77.23) | 11.3 (122.96) | 2.1 (34.93) | 0.5312 |
|  | 6 Months | Mean (SD) | -29.7 (167.68) | 28.8 (59.67) | -4.6 (43.78) | 0.4930 |
| 50% or More Reduction in Symptom Number | 3-6 Weeks | n (%) | 11 ( 73.3) | 7 ( 43.8) | 1 ( 25.0) | 0.1354 |
|  | 6 Months | n (%) | 6 ( 46.2) | 6 ( 50.0) | 0 | 0.2656 |
| 80% or More Reduction in Symptom Number | 3-6 Weeks | n (%) | 6 ( 40.0) | 6 ( 37.5) | 0 | 0.4105 |
|  | 6 Months | n (%) | 3 ( 23.1) | 3 ( 25.0) | 0 | 0.6966 |
| Resolution of Severity of Symptoms: PROMIS^®^ |  |  |  |  |  |  |
| PROMIS^®^ normalized anxiety value (3) | 3-6 Weeks | n (%) | 4 (100.0) | 4 ( 57.1) | 0 | 0.2364 |
|  | 6 Months | n (%) | 4 (100.0) | 5 (100.0) | 0 | - |
| PROMIS^®^ normalized fatigue value (3) | 3-6 Weeks | n (%) | 1 ( 33.3) | 0 | 1 (100.0) | 0.6000 |
|  | 6 Months | n (%) | 1 ( 33.3) | 0 | 0 | 1.0000 |
| PROMIS^®^ normalized sleep value (3) | 3-6 Weeks | n (%) | 0 | 0 | 1 (100.0) | 0.5000 |
|  | 6 Months | n (%) | 2 ( 66.7) | 1 (100.0) | 0 | 1.0000 |
